# Exploring the Facilitators and Barriers to Physical Activity among Adults Experiencing Food Insecurity During the Healthy Food First Trial: A Qualitative Study

**DOI:** 10.1016/j.cdnut.2026.109443

**Published:** 2026-07-21

**Authors:** Robert E Anderson, Seth A Berkowitz, Patricia Knoepp, Kevin Pignone, Kyle S Burger, Carmina G Valle, Lindsey Smith Taillie, Alice S Ammerman

**Affiliations:** 1 Department of Nutrition, Gillings School of Global Public Health, The University of North Carolina at Chapel Hill, Chapel Hill, NC, United States; 2 Carolina Population Center, The University of North Carolina at Chapel Hill, Chapel Hill, NC, United States; 3 Division of General Medicine and Clinical Epidemiology, Department of Medicine, School of Medicine, The University of North Carolina at Chapel Hill, Chapel Hill, NC, United States; 4 Cecil G. Sheps Center for Health Services Research, The University of North Carolina at Chapel Hill, Chapel Hill, NC, United States; 5 Department of Health Policy and Management, Gillings School of Global Public Health, The University of North Carolina at Chapel Hill, Chapel Hill, NC, United States; 6 Monell Chemical Senses Center, Philadelphia, PA, United States; 7 Lineberger Comprehensive Cancer Center, The University of North Carolina at Chapel Hill, Chapel Hill, NC, United States; 8 Center for Health Promotion and Disease Prevention, The University of North Carolina at Chapel Hill, Chapel Hill, NC, United States

**Keywords:** food insecurity, food is medicine, physical activity, community health workers, qualitative research, intervention implementation, cardiometabolic health, health behavior change

## Abstract

**Background:**

Food insecurity (FI) is associated with lower physical activity (PA) and higher cardiometabolic risk. Food Is Medicine (FIM) interventions increasingly demonstrate improvements in diet quality and FI, yet little is known about perceived PA barriers and facilitators within these interventions.

**Objectives:**

To examine perceived barriers and facilitators to PA among adults experiencing FI during participation in a randomly assigned FIM intervention, with attention to the role of community health worker (CHW) coaching and factors shaping PA capacity.

**Methods:**

This qualitative study was embedded within the Healthy Food First randomized clinical trial among adults with hypertension and FI, in which participants were assigned to food support (produce delivery or grocery subsidy) with or without CHW lifestyle coaching. Semi-structured telephone interviews were conducted at 12 mo or 18 mo, depending on intervention duration. Transcripts were analyzed inductively using constant comparison. Themes were organized across 3 domains: PA engagement, CHW coaching experience, and health and PA context.

**Results:**

Interviews with 38 participants, most of whom were female (66%) and Black (53%), indicated that PA was important for health and quality of life. Motivation to remain active was common. Sustained engagement was constrained by time scarcity, fatigue, occupational demands, environmental safety concerns, weather, health complication history, and financial limitations. Social connection and access to PA-friendly environments were key facilitators. Occupational PA varied widely; physically demanding work often limited leisure-time PA. CHW support was viewed as encouraging, but a stronger emphasis on diet than PA constrained its influence on PA engagement. Participants also described bidirectional links between diet quality, energy, and capacity for PA.

**Conclusions:**

Among adults experiencing FI, motivation for PA was high, but engagement was constrained by contextual and capacity barriers. PA support within FIM interventions appears acceptable and warrants testing using more explicit, low-burden approaches delivered alongside nutrition-focused components.

This trial was registered at clinicaltrials.gov as NCT05048836

## Introduction

Food insecurity (FI) remains a major public health challenge in the United States, affecting ∼47 million adults despite decades of sustained federal and state efforts to reduce its prevalence [1]. FI is associated with poorer diet quality and elevated cardiometabolic risk, including higher prevalence of hypertension, obesity, and cardiovascular disease [2,3]. Physical activity (PA) is a foundational component of chronic disease prevention and management and is emphasized in national guidelines for its cardiometabolic benefits; however, in contrast to diet quality and clinical outcomes, the relationship between FI and PA remains less well understood [4,5]. The limited literature examining FI and PA has reported mixed and, at times, paradoxical findings, with some studies observing lower leisure-time or total PA among adults with FI compared with food-secure adults, and others reporting similar levels or no significant differences [6,7]. These inconsistencies likely reflect the heterogeneous factors that shape PA, including occupational demands, time constraints, environmental conditions, health status, and access to supportive resources [[8], [9], [10]].

Since 2022, food is medicine (FIM) interventions, including medically tailored foods, produce prescriptions, and grocery subsidies, have been implemented across clinical and community-based settings and have demonstrated improvements in food security, diet quality, and cardiometabolic outcomes [[11], [12], [13]]. Within FIM interventions, community health workers (CHWs) are increasingly integrated as trusted, relationship-based supports who help participants translate nutrition guidance into daily practice and navigate barriers related to food security and health management [14,15]. Despite the growing emphasis on exercise is medicine (EIM) and the well-established role of PA in chronic disease prevention and management, relatively few FIM interventions have meaningfully integrated PA-focused or EIM-aligned components [16]. Although CHW-supported FIM interventions have demonstrated benefits for food security and diet-related outcomes [17], their role in shaping PA engagement within FIM interventions remains poorly characterized.

The Healthy Food First (HFF) randomized clinical trial tested food access supports with or without CHW-delivered lifestyle coaching among adults with hypertension and FI. HFF focused on FI and blood pressure outcomes, with all intervention combinations improving blood pressure control, modestly greater reductions observed in the food subsidy arm compared with the food box arm, and no additional benefit from lifestyle coaching or longer intervention duration [18]. PA was assessed as a secondary outcome via self-report; however, these measures were not designed to capture perceived capacity, contextual constraints, or lived experiences that shape PA engagement.

Recognizing that qualitative inquiry is well-suited to illuminate contextual influences and lived experiences not fully captured through quantitative measures [19], a qualitative component was planned a priori to better understand how participants experienced the FIM intervention and how health, environmental, and structural factors shaped PA engagement.

Prior qualitative work in the FIM field has largely focused on food security, diet quality, and program acceptability; relatively few studies have examined perceived barriers and facilitators to PA, particularly within the context of randomized nutrition-focused interventions. Understanding PA within these interventions is important because PA is a foundational component of chronic disease prevention and management, yet is rarely explicitly incorporated into FIM programs [[20], [21], [22]].

The study presented herein sought to further probe PA findings from the HFF trial using qualitative interviews with participants. By exploring perceived barriers and facilitators to PA, perceptions of CHW support, and the personal health and contextual factors shaping activity, this qualitative analysis aims to contextualize observed PA outcomes and to inform the design of future FIM interventions that more effectively integrate nutrition and PA support in alignment with EIM principles.

## Methods

### Trial design and parent study

HFF randomized clinical trial (NCT05048836) was conducted between 2022 and 2024 and approved by the University of North Carolina at Chapel Hill Institutional Review Board (protocol #21-0992). Full trial details and primary outcomes have been described elsewhere [18]. In brief, HFF was a 2 × 2 × 2 factorial trial; participants were randomly assigned to receive either twice-monthly produce box delivery or a monthly grocery subsidy, for 6 mo or 12 mo, and with or without CHW lifestyle coaching. The HFF cohort was characterized by substantial socioeconomic challenges and financial strain, including a mean annual household income of $42,473, 22% receiving supplemental nutrition assistance program benefits at enrollment, and nearly half reporting delayed food purchases or medications because of competing financial demands [18]. The primary outcomes of the parent trial were FI and blood pressure, with PA assessed as a secondary outcome using the validated international PA questionnaire (IPAQ)-short form, a self-reported measure of walking, moderate, and vigorous activity (R.E. Anderson III, S.A. Berkowitz, K.S. Burger, C.G. Valle, L. Smith-Tallie, A.S. Ammerman, unpublished results, 2026) [23]. PA was summarized as metabolic equivalent-minutes per week according to established IPAQ scoring procedures [24].

Participants assigned to the CHW condition were offered monthly, telephone-based lifestyle coaching delivered by trained CHWs. CHWs completed standardized training in the Med-South Lifestyle Program (MSLP) curriculum and motivational interviewing techniques prior to delivering sessions [25]. The MSLP uses a participant-centered approach grounded in goal setting, self-monitoring, and motivational interviewing [26]. The curriculum emphasizes culturally relevant Mediterranean-style dietary patterns, diet-related problem solving, navigation to food access resources, and chronic disease self-management strategies tailored to individual circumstances [27,28]. Guidance related to PA was included within the coaching framework and emphasized low-intensity, lifestyle-based movement consistent with national PA recommendations, with a focus on incremental increases in daily activity rather than structured or supervised exercise [4]. PA-related content was introduced within the monthly coaching sessions, which were delivered by telephone and occurred approximately once per month for either 6 mo or 12 mo depending on randomization. Sessions lasted ∼45–60 min and followed the MSLP curriculum. PA content was typically discussed after core FI and dietary topics were addressed and/or when participants expressed readiness to engage with movement-related goals. Participants randomly assigned to the non-CHW condition did not receive lifestyle coaching. Qualitative findings examining participants’ overall experiences with FI interventions in HFF have been reported separately, with the present study extending this work to focus specifically on PA and CHW-related experiences (R.E. Anderson III, M. Vu, K. Pignone, P. Knoepp, L.H. Taylor, A.S. Ammerman, et al., unpublished results, 2026). The aims and analytic approach for the present qualitative study were specified a priori and were preregistered on the Open Science Framework (https://osf.io/r7xnj).

### Qualitative recruitment and sampling

Participants were eligible for the qualitative study if they had completed the HFF intervention and were able to participate in a telephone interview conducted in English. Individuals who withdrew from the parent trial or did not complete the intervention were not eligible. Purposive sampling was used to recruit participants across all intervention exposure conditions, including food box compared with grocery subsidy, CHW coaching compared with no coaching, and 6-mo compared with 12-mo intervention duration, to capture a range of experiences with food security supports and CHW engagement. Recruitment also aimed to reflect the demographic and intervention characteristics of the parent trial with respect to sex and race across exposure conditions. At the conclusion of intervention contacts, participants were informed about the opportunity to participate in an interview. Those who expressed interest were contacted by a research team member not involved in intervention delivery to confirm interest and schedule an interview. Participants were not selected based on self-reported PA engagement. We sought to achieve our preregistered target sample size of 40, which was selected a priori to support thematic saturation in qualitative analysis [29].

### Interviews and data collection

Semi-structured telephone interviews occurred after completion of the intervention. Participants randomly assigned to the 6-mo intervention completed interviews at ∼12 mo after enrollment, whereas participants randomly assigned to the 12-mo intervention completed interviews at ∼18 mo after enrollment. Interviews were designed to capture participant experiences with HFF, with a specific focus on perceived barriers and facilitators to PA during the intervention period and perceptions of CHW support for behavior change. The interview guide was co-developed by the research team and led by the first author based on the trial’s conceptual framework and study aims. Guide development was informed by discussions with CHWs involved in intervention delivery, and CHWs were provided an opportunity to review the guide prior to the initiation of interviews. The guide included open-ended questions about experiences with food security supports, engagement with PA, perceived barriers and facilitators to movement, and the role of CHW coaching in supporting behavior change. Participants assigned to CHW coaching were asked additional probes regarding their experiences with coaching sessions, materials, and perceived support. Guides were standardized yet flexible, allowing participants to elaborate on topics most relevant to their experiences. The interview guide used in the present study is provided in Supplemental Appendix A. The interview guide was not formally pre-tested prior to implementation.

Before each interview, participants were read an informational script describing the study’s purpose, procedures, and recording process. Interviews lasted ∼30 min and were audio recorded with participant consent. All interviews were conducted in English. Interviews followed the guide but included probes and follow-up questions to clarify responses, explore context, and capture additional perspectives as needed [30,31].

All recordings were professionally transcribed verbatim, reviewed for accuracy, and de-identified prior to analysis. Audio files and transcripts were stored on secure, password-protected servers.

### Data analysis

Data were analyzed using an inductive thematic approach that incorporated grounded theory techniques, including constant comparison and inductive coding, to facilitate the identification of patterns across participant experiences. The objective was thematic understanding rather than formal theory generation [[32], [33], [34]]. Analysis focused on participants’ experiences with PA, CHW support, and the health and contextual factors shaping activity. The present analysis was designed to identify shared experiences across participants rather than compare themes according to coaching attendance or PA engagement. Accordingly, associations between PA measures, coaching attendance, and thematic content were not examined. Data collection and analysis proceeded concurrently until thematic saturation was achieved [35]. Saturation was defined as the point at which successive interviews no longer generated new themes, concepts, or substantive modifications to the codebook. ATLAS.ti software (version 23.2.1) was used to manage and code transcripts [36]. An initial codebook was developed based on domains reflected in the interview guide, with additional codes generated inductively as transcripts were reviewed. Two trained qualitative researchers independently coded transcripts and met regularly to discuss coding decisions, reconcile discrepancies, and refine the codebook. Following coding, data were organized into matrices and grouped thematically to identify patterns related to perceived PA barriers and facilitators, CHW support, and health and contextual influences on PA. Representative quotations were selected to illustrate key themes and recurring patterns across transcripts and are presented in the subsequent text and tables. Quotations are labeled by randomized intervention components to provide analytic context and do not include additional identifying information such as age or sex. To avoid overrepresentation, no individual participant is quoted more than once within a given theme. Reporting of findings adheres to the consolidated criteria for reporting qualitative research to ensure transparency in qualitative design, data collection, and analysis [37].

## Results

### Participant characteristics

The qualitative sample included 38 adults who had completed the HFF intervention and reflected the demographic and intervention profile of the full HFF cohort [18]. Participants were drawn evenly across food delivery and grocery subsidy arms and across CHW and non-CHW conditions, with approximately half receiving CHW-delivered lifestyle support. The sample was primarily female and predominantly Black, with most participants identifying as non-Hispanic or Latino (Table 1). After 38 interviews, no new themes or substantive modifications to the codebook emerged, indicating that thematic saturation had been achieved.

**TABLE 1:** Qualitative study participant characteristics (*n* = 38)

Treatment factor	*n* (%)
Food delivery	16 (42.1)
Food subsidy	22 (57.8)
CHW lifestyle intervention	19 (50.0)
No CHW Lifestyle	19 (50.0)
Sex	
Female	25 (65.8)
Male	13 (34.2)
Race	
Black	20 (52.6)
Not reported	1 (2.6)
White	17 (44.7)
Ethnicity	
Hispanic	2 (5.2)
Not Hispanic or Latino	36 (94.7)
Annual household income, mean (SD), $	53,665 (34,719)
Receiving SNAP at enrollment	9 (23.7)

Abbreviations: CHW, community health worker; SD, standard deviation; SNAP, supplemental nutrition assistance program.

### Overview of findings

**FIGURE 1:**
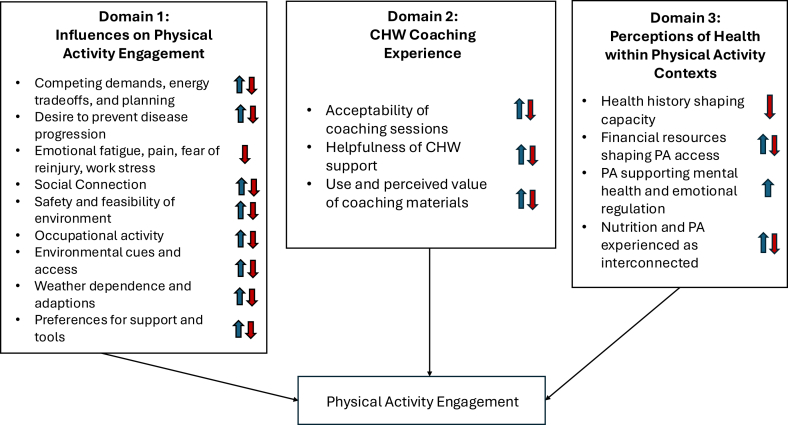
Conceptual summary of domains and themes identified through qualitative analysis. The figure summarizes the 3 domains and associated themes identified through qualitative analysis. Themes are displayed according to whether they primarily functioned as facilitators of physical activity (PA) engagement (↑), barriers to PA engagement (↓), or both facilitators and barriers (↑↓). The figure is intended to aid interpretation of relationships across domains and themes identified in this study and does not imply causal relationships. CHW, community health worker.

Across interviews, participants described PA as aspirational and important for their health, expressing a desire to engage more consistently. At the same time, engagement was shaped by competing demands, environmental and financial constraints, and health-related capacity. Figure 1 provides a conceptual summary of the relationships among the 3 domains, associated themes, and their perceived influence on PA engagement. Table 2 summarizes the domains, themes, and analytic codes identified across interviews and indicates whether each theme primarily reflected barriers to PA, facilitators of PA, or a combination of both. Themes were organized across 3 domains: influences on PA engagement, CHW coaching experience, and perceptions of health within PA contexts. Illustrative quotes are presented in the text within each theme, with additional domain and theme-specific quotations provided in TABLE 3, TABLE 4, TABLE 5 to 5.

**TABLE 2:** Domains, themes, and codes identified from qualitative analysis of participant interviews

Domain	Theme	Code: Facilitator	Code: Barrier
Domain 1: Influences on PA engagement	Competing demands, energy tradeoffs, and planning for PA	• Time for PA (planning for future routines) • Time for PA (caregiving/family routine as activity) • Time for PA (flexible schedule enabling home-based activity)	• Time for PA (time scarcity) • Time for PA (low energy after work despite available time)
Domain 1: Influences on PA engagement	Desire to prevent disease progression and stay independent	• Motivation PA (health maintenance/independence) • Motivation PA (caregiving responsibilities/family longevity) • Motivation PA (maintaining function and vitality with age)	• Motivation PA (stress and unhealthy eating cycles)
Domain 1: Influences on PA engagement	Emotional fatigue, pain, fear of reinjury, and work stress	—	• Hard to PA
Domain 1: Influences on PA engagement	Social connection supports PA engagement	• Social support PA (enjoyment with others; walking partners; family routines) • Social support PA (caregiving roles as social movement)	• Social support PA (loss of accountability partner or shared routine)
Domain 1: Influences on PA engagement	Safety and feasibility of the PA environment	• PA environment safety (inclusive and welcoming environments)	• PA environment safety (lack of sidewalks; rural hazards; terrain; traffic) • PA environment safety (racialized safety concerns)
Domain 1: Influences on PA engagement	Occupational activity shapes PA patterns	• Occupational PA (high-movement jobs; physically demanding work contributing substantial activity)	• Occupational PA (sedentary work limiting movement) • Occupational PA (fatigue after shifts, reducing leisure-time PA)
Domain 1: Influences on PA engagement	Environmental cues and access to PA	• Environment encourages PA (parks, classes, walkable destinations, supportive design, body diversity)	• Environment encourages PA (limited cues or role models; loss of subsidized access)
Domain 1: Influences on PA engagement	Weather dependence and adaptations	• Weather PA (indoor workarounds; timing strategies; gym use when accessible)	• Weather PA (heat, rain, seasonal darkness, lack of indoor options)
Domain 1: Influences on PA engagement	Preferences for PA support and tools	• Helpful for PA (goal setting, accountability, encouragement, step tracking, affordability-sensitive supports)	• Not helpful for PA (misaligned incentives or tools; redundancy; mental health concerns; unused equipment)
Domain 2: CHW coaching experience	Acceptability of coaching sessions	• Coaching sessions (session length acceptable)	• Coaching sessions (scheduling conflicts; competing demands; irregular work hours)
Domain 2: CHW coaching experience	Helpfulness of CHW support	• Coaching assistance (informative and supportive guidance; confidence building; encouragement; habit formation)	• Coaching assistance (desire for asynchronous or tech-enabled options) • Coaching assistance (limited time for PA content; perceived emphasis on diet)
Domain 2: CHW coaching experience	Use and perceived value of coaching materials	• Coaching materials (tracking and goal setting; reframing PA; recovery-oriented use; desire for PA-specific videos)	• Coaching materials (underuse outside sessions; redundancy with routines; low recall of video resources)
Domain 3: Perceptions of health within PA contexts	Health history shaping PA capacity		• Health history
Domain 3: Perceptions of health within PA contexts	Financial resources shaping access to PA	• Financial effect PA (low-cost options; free facilities; subsidies or assistance enabling PA)	• Financial effect PA (gym, class, equipment, transportation, and gear costs)
Domain 3: Perceptions of health within PA contexts	PA supporting mental health and emotional regulation	• Mental health PA	
Domain 3: Perceptions of health within PA contexts	Nutrition and PA are experienced as interconnected	• Food and PA (improved energy and willingness to move with healthier foods)	• Food and PA (certain foods reducing comfort or motivation; perceived emphasis on food over PA)

Abbreviations: CHW, community health worker; PA, physical activity.

**TABLE 3:** Domain 1: Influences on physical activity engagement

Theme	Illustrative quote
Motivation for physical activity	Well, I’m 45 y old, and I have a 4-y-old... I need to make sure that I stay as healthy as I can to be around for him.” (Food delivery CHW)
Structural and environmental constraints for physical activity	There are times when there is a lot of traffic, and I don’t want to be out in it... cars parked up and down my street. That makes it more difficult (Food delivery no CHW)
Social connection of physical activity	A friend and I had a standing appointment every week to either go to the gym or walk in the park, and we kind of fell off the past couple of months. But our intention is to get back to that. (Food subsidy CHW)
Occupational physical activity	My day job, my full-time job, is sedentary. But in the evening, the part-time job involves walking constantly and lifting things. (Food subsidy No CHW)
Participant physical activity support recommendations	Yes, that (encouraging text message or phone call) would be helpful. Yes. Just to know someone else besides family cares. (Food delivery CHW)

Abbreviation: CHW, community health worker.

**TABLE 4:** Domain 2: Community health worker coaching experience

Theme	Illustrative quote
CHW coaching session acceptability	She would send me links... suggesting YouTube videos or exercises I could do at home... Practical things... like taking multiple trips with laundry to add movement... It was helping me not think of it quite so badly... I think “exercise” is an evil word. (Food subsidy CHW coaching)
CHW coaching session acceptability	One challenge was... because I’m an independent contractor... a lot of my closings would be in the mornings... So those are sometimes when it was just a little challenging to get to the coach. (Food delivery CHW coaching)
CHW coaching helpfulness	The workout exercises, we didn’t really go over them. It was only 6 mo. (Food delivery CHW coaching)
CHW coaching helpfulness	They showed me what I can do (exercise), what can better me to get better, because with a double lung transplant, it takes a lot out of you to recover. (Food delivery CHW coaching)
CHW coaching helpfulness	I don’t remember talking about the activity part as much as the eating part. (Food subsidy CHW coaching)
CHW coaching material usage	The exercise chapters… because sometimes you think you got to exercise, you got to do it for an hour, and it sort of makes it more part of every day. (Food delivery CHW coaching)
CHW coaching material usage	I don’t remember using the notebook... I already have a routine... I think it can help to inspire movement, but it wasn’t something I needed. (Food subsidy CHW coaching)

Abbreviation: CHW, community health worker.

**TABLE 5:** Domain 3: Perceptions of health and physical activity context

Theme	Illustrative quote
Health history	I have lymphedema in both of my legs... once you start minimizing your salt and sugar intake, you might start seeing the swelling... go down. (Food delivery CHW coaching)
Financial effect	“I don’t do any of that (classes) because I can’t afford that. I do the free apps...” (Food subsidy no CHW coaching)
Financial effect	The YMCA (Young Men's Christian Association) was able to get me into a financial program. (Food delivery no CHW coaching)
Mental health	I feel a whole lot better. I’m not as tired. I feel like I’m more alert, too. So just the energy that it gives me... it makes me feel a whole lot better. (Food delivery CHW coaching)
Mental health	I can definitely tell the difference between the days or the weeks where I’m actually being intentional about making those movements... I can tell the difference between the stress levels and just emotionally feel better. (Food subsidy CHW coaching)
Interconnectedness of food and PA	Fruits and vegetables... it can have a domino effect... overall, it could have a lasting, positive effect. (Food subsidy no CHW coaching)
Interconnectedness of food and PA	I do feel like it helped (physical activity) in regards to me having better options, being able to have access to better options to eat. (Food subsidy no CHW coaching)

Abbreviations: app, application; CHW, community health worker; PA, physical activity; YMCA, Young Men's Christian Association.

#### Domain 1: Influences on PA engagement

Participants described motivation for PA alongside structural and environmental barriers, social influences, occupational demands, and preferences for future support.

##### Theme: Motivation

Many participants described strong motivation to be physically active in response to medical advice, to prevent disease progression, and to stay healthy for family responsibilities, reflecting a key facilitator of PA. At the same time, competing demands and emotional and physical fatigue functioned as barriers to consistent engagement, even when time was theoretically available.

*“Well, it’s very important for me now because I just want to keep that up...I’ve had my heart stent and everything, the doctor has told me I’ve been doing very well.”* (Food delivery CHW coaching)

*“If the day was stressful…I mean, I deal with younger kids, and sometimes it can be exhausting, not only just physically but mentally too, or if there’s just something going on personally.”* (Food subsidy no CHW coaching)

Participants often described time as available but practically constrained by stress, fatigue, and competing priorities.

*“Some days, with a high-stressful job, I just want to sit down and stare at some stupid stuff on the TV.”* (Food delivery no CHW coaching)

##### Theme: Structural and environmental constraints

Environmental barriers such as lack of sidewalks, rural terrain, traffic, seasonal darkness, and weather were consistently described as major constraints on PA engagement. Some participants also described racialized safety concerns that reduced comfort with outdoor activity.

*“(There’s) nothing in my neighborhood or community…we don’t have sidewalks. I’m out in the country. We have wild dogs…If I go walking, I have to have a golf club or something. No, I don’t like walking in my neighborhood.”* (Food delivery no CHW coaching)

*“I’m a Black person, so it’s just for whatever reason, I’ve just heard many bad things happen when you’re a Black person, you’re just running around out in public...I just want to be seen as I’m in a calm state.”*(Food subsidy CHW coaching)

In response to these constraints, some participants described adaptive strategies that partially mitigated barriers to PA.

*“We’ll leave after dinner and go to Lowe’s or Walmart and walk around for an hour.”* (Food subsidy no CHW coaching)

*“Depends on the weather because I don’t have an indoor gym to work out in, so I’m weather dependent... challenging when it is the summer (hot) as it has been.”* (Food delivery no CHW coaching)

##### Theme: Social connection

Participants described social connection as a facilitator of PA, noting that activity was more enjoyable and sustainable when done with others. Inclusive environments, including seeing a diversity of body sizes in activity spaces, increased comfort and willingness to engage.

*“Well, I go to the gym by myself, and that’s fine. But I do enjoy when we (wife) go for walks. We usually walk 3 or 4 times a week, and I enjoy walking with her. So I don’t like really walking by myself*.*”* (Food delivery CHW coaching)

*I see a lot of people there, especially at the track... I’m seeing all sizes of people. And just seeing that makes me a little bit more comfortable doing it.”* (Food subsidy CHW coaching

##### Theme: Occupational PA

Occupational activity functioned as both a facilitator and a barrier to PA, depending on job type and workload. Many participants described physically demanding jobs that contributed to substantial daily activity, including roles requiring prolonged standing, lifting, or constant movement. Several reported working multiple jobs, sometimes on the same day. These workloads often left participants physically depleted and reduced motivation and capacity for leisure-time PA. In contrast, participants with predominantly sedentary work acknowledged opportunities to engage in PA during the workday, such as walking breaks or nearby resources, but described challenges in engaging in these opportunities.

*“I don’t have that job right now... But I was doing ∼30,000 steps a day...cleaning activity, custodial. Yeah. So vacuuming, mopping, sweeping.”* (Food delivery no CHW coaching

*“I work a four-and-a-half-hour shift, and I am exhausted after*. *I stand on my feet and walk. I probably walked a good 3‒4 miles (at work)... I’m tired.”* (Food delivery no CHW coaching

*“It’s 90% sitting and 10% standing...But in the past 7 d, I actually took a walk with my coworker, and we ended up walking a mile. That was kind of unexpected...But because I’m so sedentary, I’m at a desk, I have access to the greenway, and I should be utilizing it way more than I am.”* (Food subsidy CHW coaching

##### Theme: Participant support recommendations

Participant recommendations highlighted both facilitators and barriers related to PA support tools, with preferences shaped by affordability, alignment with personal needs, and mental health considerations. Participants expressed interest in tools that increased accountability and guidance, such as step tracking or supportive messages, but emphasized affordability and alignment with personal preferences. Others described incentives or equipment as unhelpful or misaligned with mobility limitations or mental health concerns.

*“Just setting goals and making sure you achieve them... having somebody to hold you accountable.”* (Food subsidy CHW coaching)

*“I have depression... I don’t want to be penalized because of my mental state and not participating in a reward (challenge).”* (Food delivery no CHW coaching

*“We had 1 of those (step tracker)... it died. I wouldn’t mind having another one.”* (Food delivery no CHW coaching)

#### Domain 2: CHW coaching experience

Participants assigned to CHW coaching described the support as encouraging and informative, while also noting feasibility constraints that limited consistent engagement with coaching sessions and PA-related goals.

##### Theme: CHW coaching session acceptability

Session length was generally viewed as acceptable, functioning as a facilitator, while scheduling challenges and competing work demands were described as barriers to consistent participation.

*“Most of my issues were that I was always busy. I would forget and be working when they would call*. *It was just always me being occupied with work.”* (Food subsidy CHW coaching)

*“The length was perfect. It wasn’t overly like, oh! my God, I wish this lady would be quiet already. It was a decent length of time to get their point across.”* (Food delivery CHW coaching

##### Theme: CHW coaching helpfulness

Participants described CHW support as helpful for clarifying PA recommendations, reviewing goals, and providing practical guidance to increase activity. At the same time, some noted that the structure and timing of coaching limited consistent access to this support, prompting suggestions for more flexible or asynchronous options to better sustain PA engagement.

*“She was always really helpful when we did talk. We would go through the workbook, and that part I really liked*. *And she was always really informative about my questions.”* (Food subsidy CHW coaching)

*“Maybe if my questions for the health coach, which a chatbot could take care of, could be done in an off-time.”*(Food subsidy CHW coaching)

##### Theme: CHW coaching material usage

Use of coaching materials, including notebooks and videos, varied across participants. Some used materials for tracking and goal setting, whereas others expressed interest in additional PA-specific resources, such as guided exercise videos or home-based options.

*“(In the notebook) I kept my caloric intake, exercises I’m doing. I keep a log of any cardio that I’m doing, for how long I was running for.”* (Food subsidy CHW coaching)*“Probably to incorporate the physical part of it in the program... may provide a link for an easy workout to do things at home.”*(Food subsidy CHW coaching)

Some participants noted that coaching emphasized diet more than PA, reinforcing perceptions that nutrition was the primary focus of the intervention.

*“I don’t remember talking about the activity part as much as the eating part, though, to be honest.”* (Food subsidy CHW coaching

#### Domain 3: Perceptions of health and PA context

Participants described health status, financial resources, mental health, and nutrition as interacting factors that shaped both perceived capacity for and consistency of PA.

##### Theme: Health history drove PA patterns

Health history was most often described as a barrier to PA, shaping both perceived capacity and acceptable forms of movement. Many participants described chronic pain, mobility limitations, cardiovascular conditions, or recovery from serious health events as constraining their activity options. PA was often reframed as maintaining circulation, mobility, or function rather than achieving leisure-time fitness goals.

*“I’m limited to just getting up, sitting down, taking 40 steps... So I’m just pretty much just trying to keep the blood flow through my legs.”* (Food subsidy no CHW coaching

These constraints interacted with environmental and occupational demands, further shaping what participants perceived as realistic or safe.

*“We got a lot of hills and inclines that I can’t—being as far as the terrain that with my knee pain, I’m not able to navigate.”* (Food delivery no CHW coaching)

##### Theme: Financial resources shaped accessibility to PA

Financial resources were most often described as barriers to PA, influencing access to gyms, classes, equipment, transportation, and preferred forms of activity. However, some participants described the temporary financial support provided through the intervention as facilitating PA engagement. Others adapted by relying on low-cost forms of activity, although these approaches often reflected accommodation to financial constraints rather than removal of those constraints.

*“At the beginning (of the study) it was very cost-prohibitive, but the subsidy was extremely helpful.”* (Food subsidy CHW coaching

*“Besides the things for the bicycles... it doesn’t cost me any money, except just a little bit of gas.”* (Food delivery CHW coaching

*“Well, I had to stop it because... I could not afford the membership (gym), even though it was only $10.”* (Food delivery CHW coaching

*“I have limited stuff, and I need new ones (sneakers)... they get used up pretty well.”* (Food subsidy CHW coaching

##### Theme: PA supported mental health and emotional regulation

Participants consistently described PA as a facilitator of mental health, emotional regulation, and stress management. Several described noticing clear differences in emotional well-being during periods of regular PA compared with times of inactivity.

*“I feel better, have energy when I’m physically active. And I can clear my mind sometimes because my job is very stressful.”* (Food subsidy no CHW coaching

These mental health benefits often reinforced motivation to remain active, even when physical or structural barriers were present.

*“Well, other than the little bit of pain, if I overdo it, it makes me feel better...especially with my moods and stuff because I have suffered from depression.”* (Food delivery no CHW coaching

*“So mentally, I had gotten into maybe a depressive state, but once I began to get more active... I can really attribute that to the program, then having access to these different fruits that can physically naturally give you energy that can alleviate or diminish inflammation.”* (Food subsidy no CHW coaching

##### Theme: Nutrition and PA were experienced as interconnected

Nutrition and PA were experienced as bidirectionally linked, functioning as both facilitators and barriers depending on food quality, energy levels, and physical comfort. Access to healthful foods was perceived as improving energy and willingness to be active, while less healthful foods were associated with fatigue and reduced motivation.

*“If you don’t eat too heavy of food, you’re not as tired… the foods that you’re giving us help fuel us to give us more energy.”* (Food delivery CHW coaching)

*“What you put in your body is what you get out of it. If I make something that makes my stomach swell up, I’m not gonna want to go outside (for a walk).”* (Food subsidy no CHW coaching

*“Since I’m not eating the foods that make me feel like I’m tired all the time, it makes me want to go to the gym.”* (Food delivery coaching)

## Discussion

Despite recent advances, substantial gaps remain in understanding how and why PA responds variably within FIM interventions, highlighting the need for qualitative approaches that can elucidate lived experience and structural constraints not captured by quantitative analyses alone [38].

The purpose of this qualitative analysis of the HFF trial was to examine perceived barriers and facilitators to PA among adults participating in a multifactorial FIM intervention, with particular attention to CHW coaching support and implications for future intervention design. Across interviews with 38 participants, PA was consistently viewed as relevant for health and disease management, and many participants expressed motivation to be active and awareness of its importance. However, sustained PA engagement was constrained by interacting barriers, including time scarcity, fatigue, occupational demands, environmental safety concerns, health limitations, and financial constraints. These findings help explain why improvements in FI alone may not translate into meaningful increases in PA.

Participants’ experiences highlighted that motivation alone was insufficient to sustain PA in the absence of feasible opportunities. Even when motivation was high, competing demands, emotional fatigue, and structural constraints frequently limited sustained PA. Environmental factors, including lack of sidewalks, rural terrain, traffic, and concerns about personal safety, further narrowed available activity options, particularly for participants without access to indoor facilities. These constraints were often intensified by weather and seasonal conditions.

Occupational demands emerged as a key determinant of PA engagement. Participants in physically demanding jobs described substantial daily movement but limited remaining capacity for leisure-time PA, consistent with prior evidence linking occupational activity to reduced engagement in health-oriented exercise [39]. In contrast, participants with predominantly sedentary work recognized opportunities for PA during the workday but struggled to translate these moments into consistent behavior. These findings align with prior work by Holtermann et al. [40,41] and others examining the occupational–leisure PA paradox, in which work-related activity either substitutes for or undermines capacity for leisure-time PA [[40], [41], [42], [43]]. These findings suggest that future FIM interventions should avoid uniform PA prescriptions and instead tailor them based on total daily activity load and recovery needs. Social connection and access to supportive environments functioned as important facilitators of PA. Participants more often described sustained activity when it occurred with family members or in low-barrier settings such as apartment gyms, public parks, or school tracks. In these contexts, PA was integrated into existing routines rather than approached as a separate or discretionary behavior.

Across interviews, CHW lifestyle coaching was perceived as encouraging and informative, which is consistent with prior evidence supporting the role of CHWs in facilitating health behavior change in priority populations [44]. CHW coaching facilitated engagement with PA and related health behavior change by increasing accountability, self-efficacy, and perceived support. At the same time, participants frequently described scheduling challenges and competing work and family demands as barriers to consistent engagement. Many also perceived a stronger emphasis on dietary counseling than on PA guidance, reflecting the structure and priorities of the HFF coaching curriculum. These findings indicate that PA support delivered through CHWs is acceptable and welcomed within an FIM intervention. CHWs have demonstrated effectiveness in supporting chronic disease self-management and have been incorporated into PA-focused interventions, suggesting they are well-positioned to support PA when such content is intentionally integrated [[45], [46], [47]]. Many of these interventions have employed established behavior change techniques, including goal setting, self-monitoring, and problem solving, which are well supported in the PA literature [48]. However, these strategies have not been systematically evaluated within FIM-based CHW delivery models. Our findings point to the need for more deliberate alignment of FIM and EIM strategies. CHWs may serve as a practical bridge to operationalize this integration within existing health systems. Current evidence supports that embedding low-burden PA goal setting, self-monitoring, and problem-solving alongside nutrition counseling may better support PA engagement when tailored to individual health status, work demands, and environmental context [49,50]. In practice, this could include brief PA “snacks” anchored to routine activities (e.g., after-dinner walking, stair bouts, use of local tracks), targeted text messages to support problem-solving around common barriers such as safety and weather, and modalities matched to occupational load. For example, individuals in physically demanding jobs may benefit from recovery-focused modalities, whereas individuals in predominantly sedentary jobs may benefit from break-based prompts and opportunities to interrupt prolonged sitting [51]. Future CHW interventions should test PA prescriptions that vary in intensity in relation to available time and occupational demands, recognizing that higher-intensity options, when appropriate, may help increase cardiometabolic health, especially as time budgets decrease.

Participants’ descriptions of health status, mental health, and financial constraints further clarify how capacity for PA is shaped by cumulative demands rather than motivation alone. Notably, PA was commonly framed as a means of maintaining function, mobility, and emotional well-being rather than pursuing fitness or performance goals. Participants also described a perceived bidirectional relationship between diet quality and PA, noting that improved access to healthful foods (e.g., through enhanced food security during the intervention) increased energy and willingness to be active, whereas heavier or refined foods contributed to fatigue and reduced motivation. This framing suggests that FIM interventions may have a distinct opportunity to leverage nutrition-related improvements to support PA engagement. Ensuring that food access supports, CHW coaching, and educational materials explicitly connect improved food security to energy, recovery, and functional capacity may help align programs more closely with the goal of supporting an “active and healthy life.” Such integration could strengthen the alignment of FIM and EIM strategies, particularly when tailored to participants’ health history, daily demands, and personal goals.

Findings from this qualitative analysis also align with emerging quantitative evidence indicating that PA differences associated with FI may be amplified by mental health status. Recent analyses of 2022 National Health Interview Survey data demonstrated that adults with low food security reported significantly lower moderate-to-vigorous PA across all levels of depressive symptoms, with the largest absolute differences in moderate-to-vigorous PA observed among those with severe depressive symptoms compared with food-secure adults (R.E. Anderson III, M.E. Wende, J. Stroope, L.E. Balis, K. Goins, C. Bridges Hamilton, et al., unpublished results, 2026). Notably, even among individuals without depressive symptoms, low food security was associated with substantially lower PA. Considered alongside the present findings, these data suggest that FI-related constraints on PA extend beyond motivation alone and reflect cumulative effects of psychological distress, fatigue, and reduced functional capacity [52,53]. Although participants in the HFF trial experienced improvements in FI, the cohort did not consistently achieve full food security, a distinction that may be consequential [18]. Persistent FI, even when reduced, may continue to shape daily tradeoffs, energy availability, and perceived feasibility of PA, particularly when compounded by depressive symptoms [54,55]. These findings underscore that improvements in FI alone may be insufficient, and that the magnitude and sustainability of food security improvement should be considered when designing and evaluating PA within future FIM interventions.

Several limitations warrant consideration when interpreting these findings and applying them to future FIM intervention designs. First, interviews were conducted only with participants who completed the HFF intervention and agreed to qualitative follow-up. Individuals who disengaged earlier or declined interviews may have experienced different or more severe barriers to PA, including greater time constraints, health limitations, or competing stressors. As a result, the domains and themes identified here may underrepresent the experiences of participants facing the greatest challenges to PA engagement. Additionally, participants assigned to the longer intervention duration may have experienced a longer recall window for earlier intervention experiences, although interviews were conducted after completion of each participant’s assigned intervention period. Second, although the qualitative sample reflected the demographic and intervention characteristics of the parent HFF trial, HFF was a single 2 × 2 × 2 factorial FIM study conducted in the southeastern United States. Occupational demands, environmental conditions, and safety concerns described by participants are therefore context-dependent and may differ in regions with denser or sparser infrastructure, different employment or health systems, and/or alternative models of FIM delivery.

Third, this qualitative analysis focused on participants’ perceptions of PA rather than incorporating objective or self-reported quantitative PA measures into thematic interpretation. As a result, we are unable to determine how the described barriers and facilitators corresponded to measured activity levels [56]. Fourth, the CHW coaching curriculum emphasized food security and diet quality more consistently than PA. As a result, participants’ interview responses may reflect differential emphasis within the intervention rather than the full scope of their PA experiences outside the program. Finally, because most participants experienced reduced but persistent FI, these findings reflect PA barriers and facilitators under ongoing constraint rather than during a period of full food security [18].

In conclusion, this qualitative analysis identifies key barriers and facilitators shaping PA engagement among a subsample of FI hypertensive adults participating in a FIM intervention and offers actionable insight for future intervention design. Participants were motivated to be active and generally understood the health relevance of PA. What constrained engagement was capacity: fatigue, pain, time scarcity, occupational demands, safety concerns, and financial limitations. These constraints operated through daily demands rather than a lack of motivation. Social support, access to low-barrier environments, and supportive coaching facilitated PA when aligned with participants’ routines and constraints. CHWs were trusted and perceived as effective, yet PA guidance was secondary by design rather than by participant preference. Taken as a whole, facilitators such as social support, access to low-barrier environments, and supportive CHW coaching were likely insufficient to overcome the constraints participants faced, helping to contextualize prior findings that moderate improvements in FI were not associated with increases in PA.

Future FIM interventions should not assume that improvements in FI will automatically translate into increased PA. Instead, PA support should be explicit, low-burden, and tailored to the whole-person context, including occupational load, health status, environment, and the degree and sustainability of food security achieved. Integrating EIM strategies in this way offers a more realistic and testable path for supporting PA as part of cardiometabolic health within FIM interventions.

## Author contributions

The authors’ responsibilities were as follows – REA, ASA, SAB: designed the research; REA, PK, KP: conducted the research; REA, PK, KP: analyzed the data and performed statistical analysis; REA, PK, KP, ASA, SAB, LST, CGV, KSB: contributed to the writing of the paper; REA: had primary responsibility for final content; and all authors: read and approved the final manuscript.

## Data availability

Data described in the manuscript, code book, and analytic code will not be made available because the terms of our Data Use Agreement do not permit sharing of individual patient data.

## Declaration of Generative AI and AI-assisted technologies in the writing process

The author(s) declare that no generative AI or AI-assisted technologies were used in the writing of this manuscript.

## Funding

This work was supported by Blue Cross Blue Shield of North Carolina contract number UNI-3001842-SOW8. The funders had no role in the design of the study; in the collection, analyses, or interpretation of data; in the writing of the manuscript; or in the decision to publish the results.

## Conflict of interest

SAB reports research grants from NIH, the North Carolina Department of Health and Human Services, the American Heart Association, the American Diabetes Association, and Feeding America, and personal fees from the Aspen Institute, the Rockefeller Foundation, the Gretchen Swanson Center for Nutrition, and Kaiser Permanente, outside of the submitted work. All other authors report no conflicts of interest.
