# Supplementary material for: Exploring the Facilitators and Barriers to Physical Activity among Adults Experiencing Food Insecurity During the Healthy Food First Trial: A Qualitative Study
Source: Curr Dev Nutr. 2026 Jul 21;10(8):109443. doi: 10.1016/j.cdnut.2026.109443 (PMC13475409; doi:10.1016/j.cdnut.2026.109443)
Supplement: Supplementary file 1 [file mmc1.docx]

**Supplementary Materials**

**Appendix A.**

**Healthy Food First (PI: Dr. Darren DeWalt)**

**Participant Interview Guide- Physical Activity**

**Introduction**

**Greeting:** Hello, my name is _________ and I work with the research team at University of North Carolina at Chapel Hill on the Healthy Food First program. I really appreciate you taking the time to participate in this interview.

**Purpose:** The purpose of this interview is for me to learn about your experiences with the Healthy Food First program and get your suggestions for making it better.

**Confidentiality and Introduction:** You should feel free to make any sort of comments – positive or negative – about what we are talking about today. **Before we begin, I would like to remind you that your participation in this interview is voluntary. If you choose not to participate, it will not affect your care in any way, and you may still participate in other aspects of the study**. There are no right or wrong answers. Your participation is voluntary, and we can skip any question you do not want to answer. Our discussion today will last about **30 minutes**.

**Also, we record these interviews so they can be transcribed and analyzed. Is it ok to record this interview?**

**(End interview if participant declines)**

As a thank you for your time and participation, we will send you a $50 gift card.

Do you have any questions before we begin?

**START RECORDERS**

**Section 1. Overall Program Experience**

To start off, I would like to learn a little bit about your overall experience with this program.

1. What are some reasons you wanted to participate in this program?
2. What concerns, if any, did you have about participating in the program?
3. Based on your experiences, what would you tell someone who is about to begin this program?

**Section 2. Program Intervention Components**

(Ask the following sections as relevant): As part of the program, you also received:

**Health Coaching**. Tell me about your experience with the health coach.

1. How did you feel about the length of the sessions you had with your health coach?
2. Everyone is busy, which may makes it hard to schedule the sessions. What made it challenging for you to schedule the sessions?

What ideas do you have for how to make it work better?

3. In what ways did working with your health coach help you?
   a. What did you feel about the topics discussed in the coaching sessions? if needed: what topics helped most? What topics were less important to you?
   b. "In what ways, if at all, did the coaching help you identify ways to afford a healthy diet?
4. How could we make the health coaching more helpful to you?
5. What did you find useful about the Healthy Food First notebook?
   a. To what extent, if at all, did you use the notebook between the times you met with the health coach?
6. If you had the chance to view any of the videos, what was helpful or not helpful about them?

Overall, what are your thoughts about the **recipe book**?

a. Did you end up using any of the recipes?
b. Would you continue using any of the recipes?
c. What could we do to make the recipe book better?

Do you remember talking with your health coach about being physically active?

**Prompt:** Could you say a little about what you remember about that?

**Prompt:** Did you use the Healthy Food First notebook for physical activity ideas?

**Section 3. Food Self Efficacy**

I’d like to shift gears and talk about your thoughts about food.

1. Looking back, what changes, if any, did this program have on your idea of a healthy meal?
2. As part of this program, you may have tried different foods. What would you say was the biggest difference compared to the foods you usually or normally eat?
   a. To what extent do you feel you have continued to eat these different food items?
   b. What difference, if any, did the program make in terms of your food budget?
3. Thinking about your experiences with providing healthy foods for your family. What impact, if any, has participating in the program had on the foods you currently prepared?
4. Have you continued to buy and eat new foods that you might have learned about during the program?, if so, which ones? if not, why not?

**Section 4. Physical Activity**

**Individual**

To start this next portion, I'd like to learn a little bit about your health, so let me start with 3 brief yes/no questions.

1. Has your doctor ever said that you should only do physical activity when observed by a health professional?

2. Diagnosed with arthritis or osteoarthritis?

3. Any major injuries, back pain, or concerns about your balance?

For this interview, when we talk about physical activity, we mean any body movement that works your muscles and requires more energy than resting or sitting.

1. What types of physical activity do you enjoy?

2. What are some examples of physical activities that you do?

3. How do you feel when you are physically active?

**Occupational PA and PA Health Paradox**

Are you currently working outside the home or working from home? *If yes, proceed with Occupational PA question. If* *no, proceed to the* *Time question.*

Thinking about the past 7 days, how would you describe physical activity during your typical workday? *This involves only your workday, and does not include travel to and from work, or what you did in your time outside of work.*

What percentage of the day would you say you spend:

a. Sitting *including driving that is part of your job*
b. Standing
c. Walking
d. Doing heavy labor or demanding physical tasks

**Leisure Time PA**

Now I’d like to talk about non-work related physical activity.

Time

How easy is it for you to find time to be physically active?

**Prompt:** What things make it easy?

**Prompt:** What things make it difficult?

**Motivation to be physically active**

All things considered; how important is it for you to be physically active?

**Prompt:** What are the reasons being physically active is important to you?

**Motivation to be physically inactive**

What kinds of things make you *not* feel like being active?

Self-efficacy

What impact, if any, does confidence in your capability have on the kinds of physical activities you do/ or like to do?

Financial

How, if at all, does cost affect your ability to be active?

**Prompt:** Budget, gym membership, equipment? Can you tell me more about that/those?

Mental Health

Thinking about the times you are physically active, tell me what effect that has on your mood? Energy levels?

**Prompt:** Can you tell me more about that?

**Environmental**

What, if anything, about your neighborhood or nearby community helps you to be physically active?

**Prompt:** walking trails, parks, streetlights, sidewalks, walkability tracks, gyms, friends/social support,

What if anything about that/those encourages you to be physically active?

**Prompt:** cleanliness, equipment, space?

Perceived Safety

What, if anything, about your neighborhood or nearby community makes it hard to be physically active.

**Prompt**: broken or no sidewalks, crime, lighting, loose dogs, traffic, distance?

Weather

How, if at all, does the weather impact your physical activity?

**Prompt:** Can you tell me more about that? Is there a place where you can be active that is out of the weather?

Social Support (Family & Friends)

How often, if at all, do you and your family or household members participate in physical activities together? This could include going for a walk together, playing catch in the backyard, etc.

**Prompt:** What do you do? What do you think are some reasons for that?

**Prompt:** If no, what do you think are some reasons for that *not being active together*?

Food and PA

What would you say was the biggest difference having access to food had on your physical activity compared to the physical activity you usually or normally do?

Intervention Co-Design

What are some topics and/or skills you would be interested in learning that could help you to be more successful in engaging in regular physical activity?

I’m going to list several thing that help some people stay active. For each one, can you tell me how helpful each one would be for you?

**Prompt:** Can you tell me more about why you feel that way about it?

- Monthly gym allowance
- Step counter for your wrist / Wearable device
- Step/activity rewards (prizes, money, or discount on health insurance)
- Text Message or phone call encouragement
- Exercise equipment at home
- Coach/Personal trainer
- Friend to “pester’ you to exercise with them

Closing

Is there anything else about this topic that I didn’t ask that you think would be important for me to know?

**Section 5. Feelings about Health**

This program focused on foods and resources to help people be healthy in different aspects of their lives, including eating a healthy diet and managing their blood pressure.

1. Thinking about **blood pressure**, how do you feel about how you are managing your blood pressure now? Was there anything in particular that “stuck with you” from the program about how to manage your blood pressure?
2. Looking back, what impact, if any, did you feel that this program had on your **overall health**?
   a. What impact, if any, did participating in the program have on the physical activity you are doing now?
   b. Is there anything our program could have done to help you be physically active?
3. The HFF program was not focused on body weight, but we are curious whether you noticed any changes in your weight during or after the program. Has anything changed since the end of the program?

**Closing**

We talked about many different things today.

1. Looking back, what do you most appreciate about this program?
2. What is one thing that would make it better?
3. What might help someone who participated in this program continue to make positive changes in their health?

Your insights have helped us greatly. If you have any additional questions about what we have one today, do not hesitate to contact us.

Thank you!

**TURN OFF RECORDERS**
